# DMP-1 expression in alveolar bone socket following *Anredera cordifolia* (Ten.) Steenis treatment: A histological study

**DOI:** 10.5455/javar.2024.k775

**Published:** 2024-06-06

**Authors:** Christian Khoswanto, Ira Kusuma Dewi

**Affiliations:** 1 Department of Oral Biology Faculty of Dentistry, Airlangga University Surabaya, Surabaya, Indonesia; 2 Dentistry Clinic Research, Surabaya, Indonesia

**Keywords:** DMP-1, *Anredera cordifolia* (Ten.) Steenis, tooth socket, alveolar bone

## Abstract

**Objective:**

The study aimed to ascertain how *Anredera cordifolia* (Ten.) Steenis Gel affects the expression of protein dentin matrix protein-1 (DMP-1) in alveolar Wistar rats after tooth extraction.

**Materials and Methods:**

Rats were given *A. cordifolia* (Ten.) Steenis gel was in the socket after tooth extraction, and then the wound was sutured. The rats were sacrificed for 8 and 15 days following tooth extraction. The results on the 8^th^ and 15^th^ days demonstrate that the expression of DMP-1 in the treatment group is significantly higher than in the control group.

**Results:**

Expression of DMP-1 in the socket after tooth extraction on days 8 and 15 with a 400x magnification light microscope in both of the *A. cordifolia* (Ten.) Steenis gel treatment groups showed significant differences compared to the control group.

**Conclusion:**

The use of *A. cordifolia* (Ten.) Steenis gel can stimulate DMP-1 expression in alveolar bone after tooth extraction.

## Introduction

Wounds are physical injuries that usually entail the rupture or tear of a tissue, such as the epithelium membrane, as well as disruption of damaged tissue. Acute and chronic wounds to the body are two types of wounds. Chronic wounds do not heal without the use of therapeutic strategies because they are stuck in the early stages of wound healing, where inflammation is high. Acute wounds go through a complex, dynamic process that involves many different cell types and results in a healed wound [1,2].

Tooth extraction is a common treatment option in dentistry all around the world. Even if the percentage of teeth extracted has decreased due to advances in preventative and conservative dentistry, there are still a variety of reasons for tooth loss. Orthodontic therapy, advanced periodontal disease, endodontic treatment failures, trauma injuries, and prosthetic treatments are some of the reasons. The huge loss of alveolar bone following extraction can impair rehabilitation that is both useful and aesthetic with detachable or fixed prostheses, such as dental implants. When a fundamental concept of surgery is broken, the odds of developing problems grow dramatically. Complications are multifaceted, with links to the patient’s health or behaviors as well as systemic and local causes. There are two types of post-extraction complications: delayed complications and late complications. Preventing intra- and post-operative complications begins with a thorough examination of the medical history of the patient, competent surgical technique, and suitable post-operative instructions [3,4].

Immediate post-extraction problems include being unable to obtain a local anesthetic, the inability to extract the tooth, tooth or root fractures, and alveolus fractures. When a tooth can’t be healed or kept in good enough shape to maintain long-term health, function, or appearance, it is time to extract it. By affecting the capacity to masticate, communicate, and, in certain cases, socialize, a person’s quality of life is directly affected by tooth loss. Furthermore, a toothless alveolus sets off a chain of biological reactions that often result in severe anatomical alterations in the surrounding area. After extraction, alveolar ridge volume loss is a persistent process involving both horizontal and vertical decreases, according to preclinical and clinical research. When it comes to implant-supported restorations, alveolar bone ridge reduction may have a significant impact on tooth replacement. As a result, preservation of the alveolar ridge has emerged as a critical component of modern clinical dentistry [5–6].

Significant alveolar bone resorption may follow permanent tooth extraction; during 8 weeks, alterations in bone dimensions may transpire in two stages. In the first phase, there was a significant drop in the alveolar bone due to the loss of bone height in the facial area. In the second phase, there was a decline in bone thickness in the lingual and facial regions. The treatment of the alveolar bone ridge must be considered in the bone resorption that would occur following tooth extraction, according to the osteological consensus, so that the bone volume is not severely diminished. Bone grafts have been used in a multitude of ways to minimize bone resorption following tooth extraction [7–8].

Following tooth extraction, alveolar bone repair will form to fill the socket cavity. Both organic and inorganic components are used in the production of new bones. Organic matter serves a critical function in mending the alveolar bone structure at the start of new bone development. Dentin matrix protein-1 (DMP-1) is a matrix substance that is shown in alveolar bone and dentin and plays a crucial function in bone repair [9].

The *Anredera cordifolia *(Ten.) Steenis plant has numerous medicinal properties. This plant is well-known for its therapeutic powers and is frequently utilized in traditional medicine. The leaves of this plant have antibacterial and antioxidant properties and can reduce inflammation and speed the healing of wounds like hematomas, increasing bone morphogenetic protein-2 via osteoblast post-tooth extraction [10]. However, research on the expression of DMP-1 in the alveolar bone using this herbal medicine has not been thoroughly investigated.

Many treatments that use the osteoinduction ability of bone mineralization and dentin matrix have been developed to speed up bone repair. DMP-1 is presently thought to be one of the molecular activities involved in bone formation. The goal of this study is to see how *A. cordifolia* (Ten.) Steenis Gel affects the expression of protein DMP-1 in alveolar Wistar rats after tooth extraction.

## Materials and Methods

### Ethics statement

The Animal Care and Use Committees of the Airlangga University Graduate Schools of Dentistry gave their approval to this work. All animal experiments followed Airlangga University’s surgical procedures, which were done under sodium pentobarbital anesthesia, according to the Animal Experiments Guidelines, with every attempt made to reduce the animals’ suffering. Ethical clearance certificate number: 411/HRECC.FODM/IV/2023.

### Animals

Male, 8-week-old Wistar rats are a type of rats used (*n = *28). The animals were cared for at the university’s animal facility, Veterinary Faculty Airlangga University, with a 12 h light and 12 h dark cycle. There was plenty of food and drink. Rats were given *A. cordifolia *(Ten.) Steenis gel was in the socket after tooth extraction, and then the wound was sutured. The rats were sacrificed for 8 and 15 days following tooth extraction.

### Histological observation

Following tooth extraction, mandibular samples were dissected, preserved in 4% paraformaldehyde and 0.1% glutaraldehyde for 12 h at 4°C, and decalcified in a 10% Ethylenediaminetetraacetic acid solution that contains 15% glycerol for 12 h at 4°C. Hematoxylin and eosin were used to stain some of the sections, and the polyclonal DMP-1 antibody was viewed under a light microscope after the creation of 5 µm-thick serial sections (Nikon, Japan).

### Statistical analysis

To determine the significance of statistical changes in the data, a *T*-test was utilized in alveolar bone post-extraction between the intervention and the positive control.

## Results and Discussion

**Figure 1.**
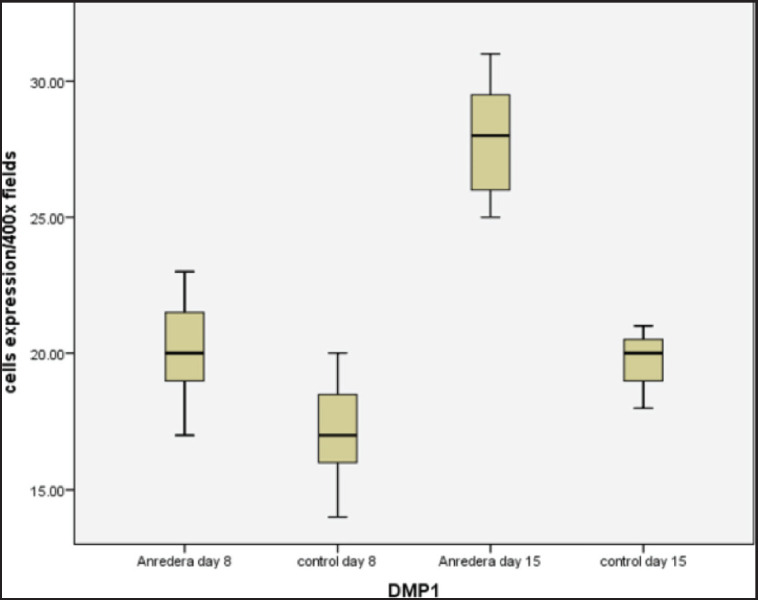
Data showing the expression of DMP1 per 400 fields in treated for 8 and 15 days.

**Figure 2.**
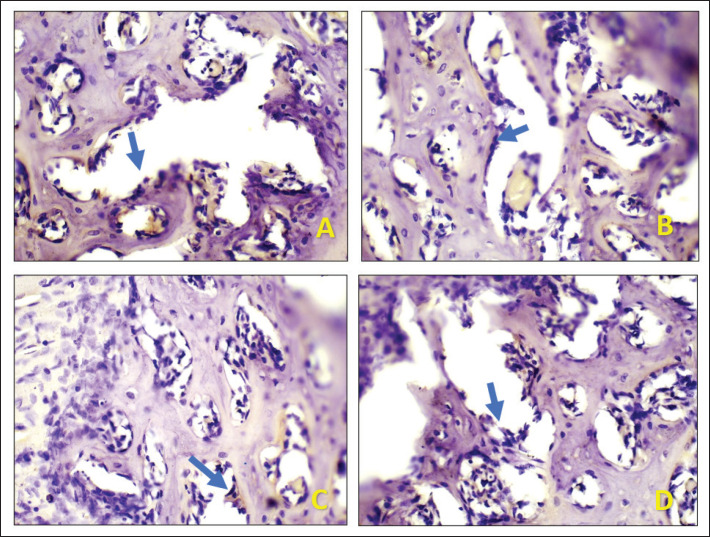
Expression of DMP-1 in socket after tooth extraction on days 8 and 15 with 400x magnification light microscope. (A) *Anredera cordifolia *(Ten.) Steenis on day 8 (B) control group on day 8. (C) *Anredera cordifolia *(Ten.) Steenis on day 15 (D) control group on day 15.

Table 1 and Figure 1 display the mean of DMP-1 expression in Wistar rats after extraction. Figures 2A and 2B show light microscopy views of the socket after tooth extraction on the 8^th^ and 15^th^ days of the *A. cordifolia* sample, respectively. Both of these light microscopy views revealed greater DMP-1 expression in comparison to the control sample (Fig. 2C–D).

Following socket extraction, a clot of the area forms, triggering an inflammatory response. At the injury’s periphery, epithelial cells will lose their desmosomal intercellular connection and move beneath the clot to establish a new epithelial surface layer. It is crucial that connective tissue be repaired and maintained on the first day of repair because it serves as a guide for forming a new surface. The clot is broken down by enzymes when the epithelial surface is restored, since it is no longer required. The regeneration of the epithelium is dependent on the repair of the basal connective tissue [11–13].

**Table 1.** The mean DMP1 expression in the treatment and control groups.

Group	X h± SD Day 8	X ± SD Day 15
Anredera K	20.14^a^ ± 2.11 17.14^b^ ± 2.01	27.85^a^ **±** 2.26 19.57^b^ ± 1.90

Note that the varied superscripts revealed a significant difference (0.05).

Vascularization occurs in the portion of bone that needs to be repaired, and mesenchymal cells develop into osteoprogenitor cells. The Golgi apparatus gets clearer, and the cytoplasm of osteoprogenitor cells transforms from eosinophilic to basophilic. This transition happens during the development of collagen tissue; sialoprotein, osteocalcin, and osseous matrix proteins are all secreted by osteoblasts. Bone matrix and osteoblasts will be involved in multiplying and attaching themselves to the cytoplasm, causing the bone matrix to calcify, while canaliculosis is the cytoplasmic protrusion of bone-producing cells that leads to the formation of osteocytes [4,14].

Permanent tooth extraction can result in considerable alveolar bone resorption; changes in bone dimensions will occur in two phases in 8 weeks. The loss in bone height in the facial area was predominant in the first phase, while the reduction in bone thickness in the facial and lingual regions occurred in the second phase, resulting in a large reduction in the alveolar bone. The alveolar bone ridge shrinks by around 50% after tooth extraction, a condition that occurs 6–12 months after the extraction of premolars and molars. This requires a drug that can withstand the rate of resorption of the alveolar bone due to tooth extraction but has a slight side effect on the body. One of the indicators of the success of the drug used in post-tooth extraction is the expression of DMP-1 in the dental extraction sockets [6,14].

DMP can be expressed both inside and outside the cell during bone development. Depending on their patterns of localization, all of these molecules have a unique function or a particular function. This knowledge could be useful in the development of bone tissue engineering strategies. According to multiple studies, DMP-1 is expressed at modest levels in diverse areas and in considerable amounts in tissues that are suffering stress, including bone. DMP-1 expression in mouse bone sections is high, with high levels in the bone and osteoblasts. The extracellular matrix (ECM)d recognizes the DMP-1 protein as two processed fragments: the N fragment of 37 kDa and the C fragment of 57 kDa [15–19].

The C component of DMP-1 is mostly located in mineralizing sites of the ECM, which contains alveolar bone and dentin tubule. The inside structure of the cell, the C segment, gathers in the mesenchymal cell nucleus. DMP-1 deficiency results in aberrant bone and dentin mineralization, resulting in hypophosphatemic rickets and osteomalacia, particularly in phenotypic individuals. According to new research, DMP-1 regulates the hormone fibroblast growth factor23 and is involved in osteoblast growth and mineralization. DMP-1 overexpression increased mineralization, and the cortical bone’s biomechanical properties were considerably altered [20–22].

Numerous investigations have shown that DMP-1 is expressed at low levels in a variety of tissues and in substantial quantities in tissues that are experiencing a contribution, such as bone and dentin. DMP-1 expression in mouse bone sections aged 3 months demonstrated elevated DMP-1 expression in both the bone and osteoblasts. Osteoclasts and osteoblasts control the two interdependent processes that mediate bone remodeling: bone resorption and bone formation. This process starts after a bone lesion and ends with the production of new bone or bone repair through bone healing. It has been suggested that DMP-1 serves as a biomarker for the progression of bone regeneration and damage repair during arthroscopic surgery [23–25]. Dmp-1 considerably increased in the alveolar bone in the base third of the socket in the present investigation following the extraction of a tooth that was given *A. cordifolia *(Ten.) Steenis treatment.

In this study, it was found that the herbal content of *A. cordifolia *(Ten.) Steenis extract was able to increase the expression of DMP-1 in wounds after the extraction of Wistar rats. This indicates that the effect of this medicinal plant can accelerate the formation of organic matrix and mineralization of alveolar bone. This is a good sign because the tooth extraction will cause significant bone resorption after a few months. It is hoped that with the administration of this drug, the resorption that occurs will be minimized.

This study identified the function of *A. cordifolia *(Ten.) Steenis extract is an herbal remedy that can raise the expression of DMP-1 in tooth extraction scars; however, because the study only lasted until day 15, it was unable to see the development of dense bone in the tooth extraction socket. To address this, more studies with a longer duration and the inclusion of protein markers involved in alveolar bone healing are required.

## Conclusion

The use of *A. cordifolia* (Ten.) Steenis gel can stimulate DMP-1 expression in alveolar bone after tooth extraction. This herb has the ability to quicken the mineralization of alveolar bone and the creation of an organic matrix. It is hoped that by using this medication, the resorption post-tooth extraction will be reduced.

## List of Abbreviations

BMP-2, Bone morphogenetic protein-2; DMP-1, Dentin matrix protein-1; EDTA, Ethylenediaminetetraacetic acid; ECM, Extracellular matrix; FGF, Fibroblast grow factor.
